# Framingham Ten-Year General Cardiovascular Disease Risk: Agreement between BMI-Based and Cholesterol-Based Estimates in a South Asian Convenience Sample

**DOI:** 10.1371/journal.pone.0119183

**Published:** 2015-03-17

**Authors:** Charlotte A. Jones, Leanne Ross, Nadia Surani, Narissa Dharamshi, Karima Karmali

**Affiliations:** 1 Department of Medicine, University of Calgary, Libin Cardiovascular Institute, Calgary, AB, Canada; 2 Faculty of Medicine, Southern Medical Program, University of British Columbia—Okanagan, Kelowna, BC, Canada; 3 Independent Researcher, Toronto, ON, Canada; University Heart Center, GERMANY

## Abstract

The goal of this analysis was to determine the agreement between body mass index-based and cholesterol-based ten-year Framingham general cardiovascular disease risk scores among a convenience sample of 773 South Asian Canadian adults attending community-based screening clinics. Scores were calculated using age, systolic blood pressure, antihypertensive use, current smoking, diabetes, and total cholesterol and high density lipoprotein (for cholesterol-based risk) or height and weight (for body mass index-based risk). Mean risk score differences (body mass index-based risk minus cholesterol-based risk) were estimated using paired t-tests. Bland-Altman plots were used to assess agreement between scores. Finally, agreement across risk categories (low [<10%], moderate [10% to <20%], high [> = 20%]) was examined using the kappa statistic. Average agreement between the two risk scores was quite good overall (mean differences of 0.6% for men and 0.5% for women), but increased to about 3% among participants 60–74 years of age. However, Bland-Altman plots revealed that the differences between the two scores and the variability of the differences increased with increasing average 10-year risk. In terms of clinical importance, the limits of agreement were reasonable for women < 60 years (95% confidence interval: -3.2% to 3.1%), but of concern for women 60-74 years (95% confidence interval: -6.0% to 12.3%), men < 60 years (95% confidence interval: -7.1% to 7.3%) and men 6-074 years (95% confidence interval: -13.8% to 18.8%). Agreement across categories was moderate for most sex and age groups examined (kappa values: 0.51 for women < 60 years, 0.50 for women 60-74 years, 0.65 for men < 60 years), except for men 60-74 years, where agreement was only fair (kappa = 0.26). In light of these disagreements, evaluation of a participant’s change in cardiovascular disease risk over time will necessitate use of the same risk score (i.e., either body mass index-based or cholesterol-based) at all screening sessions.

## Introduction

Heart disease and stroke are the second and third leading causes of death in Canada, responsible for 21% and 6% of all deaths, respectively [1]. While age-standardized mortality rates for both declined by 33% in the general Canadian population from 2000 to 2009 [1], certain groups remain at elevated risk. Despite the fact that national administrative data on cardiovascular disease (CVD) mortality and morbidity by ethnicity are not available in Canada, it has been estimated that ischemic heart disease mortality rates among men and women of South Asian (SA) origin are 3 and 3.6 times higher, respectively, than rates among men and women of Chinese origin [2]. Acute myocardial infarction hospitalization rates in British Columbia have also been shown to be higher among South Asians (SAs) compared with Chinese and Whites [3]. Internationally, the highest rates of CVD have been documented in SA countries, and risk is elevated among SAs in both their native countries and in the countries to which they have immigrated [4].

The CVD burden in these high-risk populations has prompted the development of targeted strategies to identify and manage at-risk individuals. Participating in available programs can be challenging for SAs, however, because of language barriers, cultural differences, limited health literacy, lack of knowledge about or mistrust of available service, and circumstantial challenges such as lack of transportation or financial limitations [5–7]. Community-based screening for CVD risk, shown to be feasible in a variety of SA community settings [8–10], may engage those who do not or cannot access primary care services. Continuing our pilot work [8], we have partnered with SA communities across Canada to provide a culturally appropriate, accessible and sustainable CVD screening and support program. Participants have completed baseline screening and will be re-screened after one year to assess change in CVD risk.

At baseline, Framingham general CVD risk scores were determined. The risk scores, developed by D’Agostino et al [11], are calculated using either a cholesterol-based algorithm or a BMI-based algorithm. While the Framingham risk scores have not been validated in SA populations, they can provide an estimate of 10-year absolute CVD risk for public educational purposes [11]. To provide them with as much risk factor information as possible, we aimed to assess cholesterol-based CVD risk for all participants. For some, however, only a BMI-based risk score was determined (i.e., if blood collection was declined, or if cholesterol testing supplies, which were limited due to funding constraints, ran out during any given clinic). At our one-year follow-up clinics, the same procedures will be followed. Hence, at the completion of the study, we will have a small number of participants who were assessed differently at baseline compared with follow-up. In other words, a small number will have had a cholesterol-based risk score calculated at baseline, but a BMI-based risk score calculated at follow-up (or vice versa). For these participants, it is not known if a valid assessment of change in CVD risk from baseline to follow-up can be made. For example, will an observed change in overall CVD risk, obtained by subtracting a BMI-based follow-up risk score from a cholesterol-based baseline risk score reflect a true change in CVD risk? Or will it simply reflect a discrepancy arising from the two different calculations? Answering these questions necessitates determining whether or not the two forms of the general CVD risk score yield similar estimates of overall risk for a given individual. Accordingly, the primary goal of this analysis was to assess the agreement between BMI-based and cholesterol-based CVD risk scores for participants with sufficient data to calculate both.

## Methods

### Ethics Statement

This study received ethical approval from the University of Calgary Conjoint Health Research Ethics Board (Protocol Number E-24224) and the Health Canada and Public Health Agency of Canada Research Ethics Board (Protocol Number 2011–0034). All participants provided written informed consent.

### Study Methods

The University of Calgary research team was approached by SA community leaders to implement a national program similar to three local projects that addressed CVD risk reduction [8,12,13]. The program methodology was developed collaboratively, incorporating the principles of community based participatory research [14]. At a two-day national training workshop, the research team met with the regional core teams (i.e., project managers and clinic leads) from six cities across Canada. Using a standardized, culturally-adapted training manual, the research team provided information on CVD and its major risk factors, reviewed the research methodology and screening clinic procedures, and provided hands-on training for: collection of participant health histories, measurement of height and weight, calculation of body mass index (BMI), assessment of blood pressure (BP) and capillary total and HDL cholesterol, Framingham risk score calculation, and participant counseling and referral. Using a “train the trainer” approach, the core teams returned to their respective cities to provide the same training to their local volunteer teams. All volunteers, a mix of health professionals and lay community members, were also members of the participating SA communities.

### Study Population

From February through August 2012, 792 predominantly SA participants (mostly originating from East Africa, India, Pakistan, and Central Asia), 30–74 years of age with no prior history of CVD, underwent cholesterol-based screening at community-based clinics. Ethnicity was not directly assessed in the study but we estimate that 98% of the participants were of SA origin (personal communication, N. Surani, May 9, 2014).

### Lifestyle and Health Data

Participants completed a brief health history questionnaire which assessed basic demographic data and self-report of: a prior history of heart disease or stroke; a physician diagnosis of, or prescription for, hypertension, high cholesterol, and/or diabetes; current use of any medication(s) for blood pressure, cholesterol and/or blood sugar; and current smoking status. They then underwent measurement of height, weight, blood pressure (BP) and random capillary total cholesterol (TC) and high density lipoprotein (HDL). Height and weight were measured using rigid tape measures and simple bathroom scales, purchased locally by each team. BP was measured using the BPTru (model BPM 100; VSM MedTech Ltd.), an automated device that yields a more reliable and accurate assessment of resting BP (when compared with 24-hour ambulatory measurement) than a manual measurement taken with a stethoscope and mercury sphygmomanometer [15,16]. To reduce clinic waiting times, resting BP was taken using a modification of the Canadian guidelines [17]. The BPTru monitor was set to take six readings at one minute intervals. If the first reading was normal [systolic and diastolic pressures < 140 mmHg and < 90 mmHg, respectively (if not diabetic), or < 130 mmHg and < 80 mmHg, respectively (if diabetic)], the first reading was recorded as the screening BP. Otherwise, the first reading was discarded and the average of the last five readings was recorded (as per the usual Canadian guidelines). TC and HDL were measured using the Cholestech point-of-care desktop reflometer (Hayward, CA, USA), which delivers results that correlate well with laboratory results from venous blood samples [18,19]. Ten-year CVD risk scores were then calculated and explained to participants by volunteer health professionals (licensed and practicing registered nurses, pharmacists or physicians).

### Statistical Analyses

Questionnaire and measured health data were summarized using counts and percentages (categorical data), means and standard deviations (normally distributed continuous variables) and medians, ranges and interquartile ranges (non-normally distributed continuous variables). While the clinic volunteers calculated CVD risk using the paper-based charts published by D’Agostino et al [11], for the purposes of this analysis, we calculated exact cholesterol-based and BMI-based risk scores using the risk functions derived from gender-specific Cox proportional-hazards regression models published in the same paper. The models, based on risk factors and outcomes ascertained from the original Framingham Heart Study (1968–71) and the Framingham Offspring Study (1971–75 and 1984–87), include age, systolic BP, antihypertensive use, current smoking, diabetes, and TC and HDL (for cholesterol-based risk) or BMI (for BMI-based risk). The risk functions yield an estimate of 10-year absolute CVD risk, including coronary death, myocardial infarction, coronary insufficiency, angina, ischemic stroke, hemorrhagic stroke, transient ischemic attack, peripheral artery disease and heart failure.

For each participant, the difference between the BMI-based risk estimate and the cholesterol-based risk estimate was determined (BMI-based risk minus cholesterol-based risk). Mean risk score differences and 95% confidence intervals (CIs) were estimated separately for men and women, and by the Framingham age categories used in the risk calculations, using paired t-tests.

The differences were then examined using Bland-Altman plots [20]. In a Bland-Altman plot, for each participant, the difference between the two scores is plotted against their mean. As a participant’s true CVD risk is unknown, the mean of her/his BMI-based and cholesterol-based scores is the best estimate available. The 95% limits of agreements (i.e., the mean difference between the scores +/- two standard deviations of the differences) were then estimated to provide an interval within which 95% of the differences between the two scores would be expected to lie [20].

Finally, both sets of scores were categorized as low (<10%), moderate (10% to <20%) or high (> = 20%) risk. Agreement between BMI-based risk and cholesterol-based risk across categories was examined using the kappa statistic [21].

Given that the Framingham risk functions that we used do not account for use of cholesterol medications, we repeated the analysis after excluding the 91 participants who reported hypercholesterolemia treatment. All analyses were conducted using Stata/IC 12.0 (College Stn, TX).

## Results

### Participant Characteristics

Of the 792 participants who underwent cholesterol-based risk assessment, 773 (98%) had their height and weight measured, permitting comparison of cholesterol-based CVD risk with BMI-based CVD risk. Four hundred one women (52%) and 372 men (48%) 30–74 years of age (mean: 48.9 years) were included in this analysis. The majority of participants were not born in Canada, but of those, about half had lived in Canada for longer than 20 years (Table 1). Ninety two percent selected English as their language of choice during screening. Twenty nine percent of all participants reported a first degree family history of CVD in one or more relatives before the age of 60 years. The prevalence of self-reported hypertension (physician-diagnosed) was the same among men and women (about 15%), and of those reporting the condition, 71% of women and 67% of men were being treated with medication(s). The prevalence of self-reported diabetes was similar among women and men (7.2% overall), but of those affected, women were less likely than men to report current treatment (71% vs. 92%). Women were less likely than men to report hypercholesterolemia (18% vs. 30%) and slightly less likely to report treatment if affected (45% vs. 52%). Smoking was rare overall, but more common among men than women (4% vs. 2%). On average, measured BP was slightly higher among men than women, while TC, HDL and BMI were slightly lower. Median 10-year BMI-based and cholesterol-based CVD risk scores were similar for women (5.1% and 5.2%, respectively), though the variability in BMI-based scores was higher, as evidenced by the overall and interquartile ranges. A similar trend was noted for men, who had median risk scores approximately double those of the women (12.4% for BMI-based risk and 11.7% for cholesterol-based risk).

**Table 1:** Participant characteristics among 773 South Asian Canadian adults who attended community-based screening clinics.

		Female (N = 401)	Male (N = 372)
		**mean (SD)^5^**	
Age (years)		49.5 (10.5)	48.2 (11.0)
Systolic BP^1^(mm Hg)		120.5 (15.0)	124.4 (14.2)
Diastolic BP (mm Hg)		75.6 (9.4)	80.5 (9.9)
HDL^2^ cholesterol (mmol/L)		1.34 (0.37)	1.02 (0.28)
Total cholesterol (mmol/L)		4.84 (0.86)	4.74 (0.86)
BMI^3^ (kg/m^2^)		27.1 (4.8)	26.4 (3.9)
		**median (IQR)** ^6^ **range**	
BMI-based CVD^4^ risk score (10-year, %)		5.1 (2.8–9.7) 0.6–42.1	12.4 (6.1–20.6) 1.9–65.4
Cholesterol-based CVD risk score (10-year, %)		5.2 (2.8–9.1) 0.4–31.0	11.7 (6.0–18.9) 1.3–70.1
		**n (%)**	
Age (years)
	30–39	77 (19.2)	87 (23.4)
	40–49	128 (31.9)	117 (31.5)
	50–59	116 (28.9)	105 (28.2)
	60–69	66 (16.5)	53 (14.3)
	70–74	14 (3.5)	10 (2.7)
Born in Canada
	prefer not to say	4 (1.0)	4 (1.1)
	yes	12 (3.0)	25 (6.7)
	no	385 (96.0)	343 (92.2)
Years in Canada (if not born in Canada)^1^
	prefer not to say	14 (3.6)	12 (3.5)
	<1	10 (2.6)	8 (2.3)
	1–10	74 (19.2)	71 (20.7)
	11–20	83 (21.6)	73 (21.3)
	>20	204 (53.0)	179 (52.2)
Self-report of physician-diagnosed hypertension		63 (15.7)	54 (14.5)
Current hypertension medication(s)		45 (11.2)	36 (9.7)
Self-report of physician-diagnosed hypercholesterolemia		71 (17.7)	113 (30.4)
Current hypercholesterolemia medication(s)		32 (8.0)	59 (15.9)
Self-report of physician-diagnosed diabetes		31 (7.7)	25 (6.7)
Current diabetes medication(s)		22 (5.5)	23 (6.2)
Current or recent (within 3 months) smoker		6 (1.5)	16 (4.3)
1^st^ degree relative with CVD diagnosis at < 60 years		117 (29.2)	104 (28.0)

^1^ BP = blood pressure

^2^ HDL = high density lipoprotein

^3^ BMI = body mass index

^4^ CVD = cardiovascular disease

^5^ SD = standard deviation

^6^ IQR = interquartile range (i.e., 25^th^–75^th^ percentile)

^7^ reported as percentage of those not born in Canada.

### Mean Differences in Risk Scores

The average differences between BMI-based and cholesterol-based scores for women and men were 0.6% (95% CI: 0.3% to 0.8%) and 0.5% (95% CI: 0.0% to 1.0%), respectively. When examined by the age categories used in the Framingham calculations, the average differences were larger for both women and men in the three youngest age groups (30–39, 40–49 and 50–59 years) compared with those in the two older age groups (60–69 years and 70–74 years). Accordingly, the average difference was calculated for two age groups: < 60 years and 60–74 years. For women, the mean difference between the scores for those < 60 years was-0.1% (95% CI: -0.3% to 0.1%) compared with 3.1% (2.1% to 4.2%) for those 60–74 years. For men, the same pattern was observed. Mean differences were 0.1% (95% CI: -0.3% to 0.5%) among those < 60 years and 2.5% (95% CI: 0.4% to 4.5%) among those 60–74 years.

### Bland-Altman Plots / Limits of Agreement

**Fig 1:**
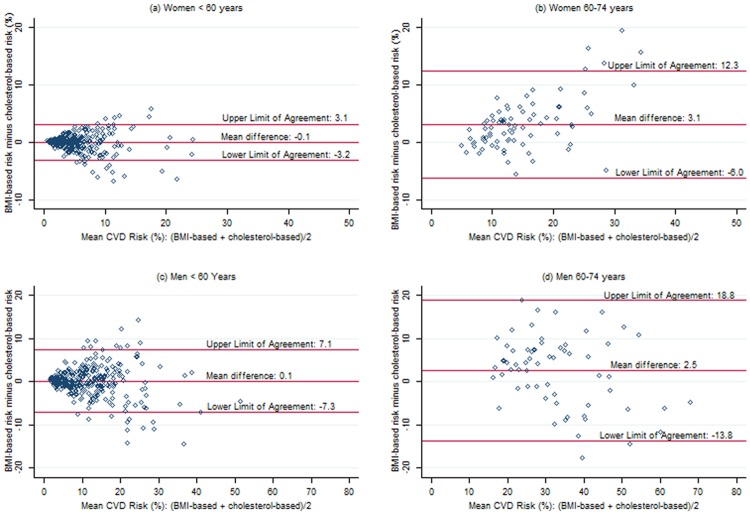
Bland-Altman plots showing agreement between BMI-based and cholesterol-based CVD risk scores. (a) Women < 60 years (b) Women 60–74 years (c) Men < 60 years (d) Men 60–74 years.

While these differences suggest that BMI-based and cholesterol-based scores agree well on average, they offer no insight into our primary goal of determining whether or not the two forms of the general CVD risk score yield similar estimates of overall risk for a given individual. Bland-Altman plots showing the agreement between the two risk scores for men and women by age group (<60 years, 60–74 years) are shown in Fig. 1. The limits of agreement shown on the plots (i.e., the mean difference +/- two standard deviations of the difference) provide estimates of the 95% range of agreement for individuals. For women, both the magnitude of the differences between the scores and the variability of the differences increase as the average 10-year CVD risk increases. The limits of agreement are reasonable in terms of clinical importance for women < 60 years (-3.2% to 3.1%), but are a concern for those > = 60 years (-6.0% to 12.3%). Similar trends were noted for men, though the limits of agreement were wide from a clinical perspective for both age groups: -7.1% to 7.3% for those <60 years; -13.8% to 18.8% for those 60–74 years.

### Categorical Agreement

Categorical agreement between the two risk scores is shown in Table 2 (women) and Table 3 (men). Overall agreement in risk category classification was 87% for women (kappa = 0.63, standard error (SE) = 0.04). While disagreements were observed in both directions, there was a tendency for BMI-based risk to be higher than cholesterol-based risk classifications. BMI-based risk categories were higher than cholesterol-based risk categories for 36 women (9%), but lower for only 18 (5%). Agreement across risk categories was 91% for women < 60 years and 69% for women 60–74 years, yielding similar kappa coefficients: 0.51 (SE = 0.05) and 0.50 (SE = 0.08), respectively. Among the younger women, disagreements were equally likely to occur in both directions. Relative to their cholesterol-based risk classifications, 15 (5%) had higher and 17 (5%) had lower BMI-based risk classifications. Among the older women, disagreements almost always reflected higher BMI-based risk; 24 (30%) had higher but only 1 (1%) had lower BMI-based risk classifications compared with their cholesterol-based classifications.

**Table 2:** Categorical agreement between cardiovascular disease risk scores among South Asian Canadian women who attended community-based screening clinics.

All Women^1^				
	BMI-Based^5^ Risk Category			
	Low	Moderate	High	Total
Cholesterol-Based Risk Category				
Low^2^	290	26	0	316
Moderate^3^	15	42	10	67
High^4^	0	3	15	18
Total	305	71	25	401

**Women < 60 Years**				
	BMI-Based Risk Category			
	Low	Moderate	High	Total
Cholesterol-Based Risk Category				
Low	276	11	0	287
Moderate	14	13	1	28
High	0	3	3	6
Total	290	27	4	321

**Women 60–74 Years**				
	BMI-Based Risk Category			
	Low	Moderate	High	Total
Cholesterol-Based Risk Category				
Low	14	15	0	29
Moderate	1	29	9	39
High	0	0	12	12
Total	15	44	21	80

^1^ The cross-tabulation of categorized 10-year Framingham general cardiovascular disease risk scores. Cholesterol-based risk score categories are listed on the left; BMI-based risk scores are listed across the top. The cells showing agreement (i.e., where both BMI-based risk and cholesterol-based risk are classified as “low”, “moderate” or “high”) run diagonally in the table. The “disagreements” are in all of the other cells.

^2^ Low risk: < 10%

^3^ Moderate risk: 10% to < 20%

^4^ High risk: > = 20%

^5^ BMI-based = body mass index-based

**Table 3:** Categorical agreement between cardiovascular disease risk scores among South Asian Canadian men who attended community-based screening clinics.

All Men^1^				
	BMI-Based^5^ Risk Category			
	Low	Moderate	High	Total
Cholesterol-Based Risk Category				
Low^2^	137	28	0	165
Moderate^3^	15	85	22	122
High^4^	0	11	74	85
Total	152	124	96	372

**Men < 60 Years**				
	BMI-Based Risk Category			
	Low	Moderate	High	Total
Cholesterol-Based Risk Category				
Low	137	28	0	165
Moderate	15	82	11	108
High	0	10	26	36
Total	152	120	37	309

**Men 60–74 Years**				
	BMI-Based Risk Category			
	Low	Moderate	High	Total
Cholesterol-Based Risk Category				
Low	0	0	0	0
Moderate	0	3	11	14
High	0	1	48	49
Total	0	4	59	63

^1^ The cross-tabulation of categorized 10-year Framingham general cardiovascular disease risk scores. Cholesterol-based risk score categories are listed on the left; BMI-based risk scores are listed across the top. The cells showing agreement (i.e., where both BMI-based risk and cholesterol-based risk are classified as “low”, “moderate” or “high”) run diagonally in the table. The “disagreements” are in all of the other cells.

^2^ Low risk: < 10%

^3^ Moderate risk: 10% to < 20%

^4^ High risk: > = 20%

^5^ BMI-based = body mass index-based

Overall agreement in risk category classification was 80% for men (kappa = 0.69, SE = 0.04). As with women, there was a tendency for BMI-based risk to be higher than cholesterol-based risk classifications. BMI-based risk categories were higher than cholesterol-based risk categories for 50 men (13%) and lower for only 26 (7%). Agreement across risk categories was 79% for men < 60 years (kappa = 0.65, SE = 0.04). Among the younger men, 39 (13%) had higher and 25 (8%) had lower BMI-based risk classifications relative to their cholesterol-based classifications. None of the men 60–74 years were classified as low risk by either scoring system. Overall agreement among the moderate and high risk categories was 81% (kappa = 0.26, SE = 0.10). Eleven (17%) classified as moderate risk according to their cholesterol-based measures were considered high risk based on their BMI-based scores, while 1 (2%) classified as high risk according to his cholesterol-based score was considered moderate risk based on his BMI-based score.

### Exclusion of Participants Reporting Hypercholesterolemia Treatment

**Fig 2:**
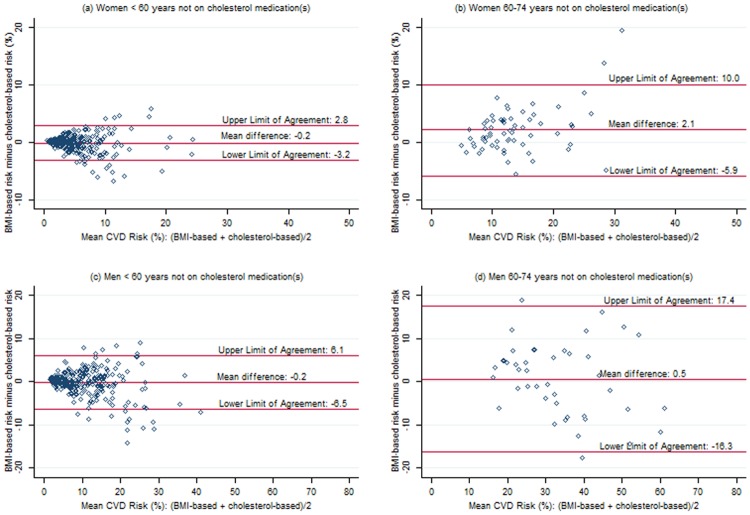
Bland-Altman plots showing agreement between BMI-based and cholesterol-based CVD risk scores for participants not on cholesterol medication(s). (a) Women < 60 years (b) Women 60–74 years (c) Men < 60 years (d) Men 60–74 years.

As expected, when the 91 participants on cholesterol-lowering medication(s) were excluded from the analysis, the mean differences between BMI-based and cholesterol-based risk scores decreased slightly, most noticeably among older participants who were more likely to be on treatment. For women, the mean differences were 0.2% (95% CI: 0.0% to 0.5%) overall, -0.2% (95% CI: -0.3% to 0.0%) for those < 60 years, and 2.1% (95% CI: 1.1% to 3.1%) for those 60–74 years. For men, the mean differences were-0.1% (-0.6% to 0.4%) overall, -0.2% (95% CI: -0.6% to 0.2%) for those < 60 years, and 0.5% (95% CI: -2.0% to 3.1%) for those 60–74 years. For both men and women across both age groups, however, the limits of agreement on the Bland-Altman plots were essentially unchanged (Fig. 2). Again, the limits of agreement were reasonable for women < 60 years (-3.2% to 2.8%), but unacceptable from a clinical perspective for women 60–74 years (-5.9% to 10.0%), men < 60 years (-6.5% to 6.1%) and men 60–74 years (-16.3% to 17.4%). Categorical agreement (Tables 4 and 5) as assessed with the kappa coefficients demonstrated limited improvement: women < 60: kappa = 0.66 (SE = 0.04); women 60–74: kappa = 0.59 (SE = 0.09); men < 60: kappa = 0.69 (SE = 0.05); men 60–74: kappa = 0.29 (SE = 0.13).

**Table 4:** Categorical agreement between cardiovascular disease risk scores among South Asian Canadian women not on cholesterol medication(s) who attended community-based screening clinics.

All Women^1^				
	BMI-Based^5^ Risk Category			
	Low	Moderate	High	Total
Cholesterol-Based Risk Category				
Low^2^	280	20	0	300
Moderate^3^	15	37	3	55
High^4^	0	2	12	14
Total	295	59	15	369

**Women < 60 Years**				
	BMI-Based Risk Category			
	Low	Moderate	High	Total
Cholesterol-Based Risk Category				
Low	267	7	0	274
Moderate	14	11	1	26
High	0	2	3	5
Total	281	20	4	305

**Women 60–74 Years**				
	BMI-Based Risk Category			
	Low	Moderate	High	Total
Cholesterol-Based Risk Category				
Low	13	13	0	26
Moderate	1	26	2	29
High	0	0	9	9
Total	14	39	11	64

^1^ The cross-tabulation of categorized 10-year Framingham general cardiovascular disease risk scores. Cholesterol-based risk score categories are listed on the left; BMI-based risk scores are listed across the top. The cells showing agreement (i.e., where both BMI-based risk and cholesterol-based risk are classified as “low”, “moderate” or “high”) run diagonally in the table. The “disagreements” are in all of the other cells.

^2^ Low risk: < 10%

^3^ Moderate risk: 10% to < 20%

^4^ High risk: > = 20%

^5^ BMI-based = body mass index-based.

**Table 5:** Categorical agreement between cardiovascular disease risk scores among South Asian Canadian men not on cholesterol medication(s) who attended community-based screening clinics.

All Men^1^				
	BMI-Based^5^ Risk Category			
	Low	Moderate	High	Total
Cholesterol-Based Risk Category				
Low^2^	132	21	0	153
Moderate^3^	13	73	10	96
High^4^	0	10	54	54
Total	145	104	64	313

**Men < 60 Years**				
	BMI-Based Risk Category			
	Low	Moderate	High	Total
Cholesterol-Based Risk Category				
Low	132	21	0	153
Moderate	13	71	4	88
High	0	9	20	29
Total	145	101	24	270

**Men 60–74 Years**				
	BMI-Based Risk Category			
	Low	Moderate	High	Total
Cholesterol-Based Risk Category				
Low	0	0	0	0
Moderate	0	2	6	8
High	0	1	34	35
Total	0	3	40	43

^1^ The cross-tabulation of categorized 10-year Framingham general cardiovascular disease risk scores. Cholesterol-based risk score categories are listed on the left; BMI-based risk scores are listed across the top. The cells showing agreement (i.e., where both BMI-based risk and cholesterol-based risk are classified as “low”, “moderate” or “high”) run diagonally in the table. The “disagreements” are in all of the other cells.

^2^ Low risk: < 10%

^3^ Moderate risk: 10% to < 20%

^4^ High risk: > = 20%

^5^ BMI-based = body mass index-based.

## Discussion

In our convenience sample of largely SA participants in a national screening program, average agreement between BMI-based and cholesterol-based 10-year general Framingham CVD risk scores at the group level was quite good overall, with average differences of 0.6% for women and 0.5% for men. These increased to about 3% among participants 60–74 years of age. Bland-Altman plots illustrating agreement between scores for individuals, however, revealed that both the differences between the two scores and the variability of the differences increased as the average of the two scores increased. In terms of clinical importance, the limits of agreement were reasonable for women < 60 years (-3.2% to 3.1%), but of concern for women 60–74 years (-6.0% to 12.3%), men < 60 years (-7.1% to 7.3%) and men 60–74 years (-13.8% to 18.8%). Considered from the perspective of low (<10%), moderate (10% to <20%) and high (> = 20%) risk, which is what our participants were counseled on, agreement as assessed by the kappa statistic was moderate to good among all sex and age groups examined, except for men 60–74 years, where agreement was only fair [22]. Limiting the analysis to those not on medication(s) for hypercholesterolemia did not appreciably change the observed agreement.

In our study, practical and budget considerations prevented us from standardizing the measurement of height and weight. Volunteers purchased bathroom scales and tape measures locally, and it was not possible to calibrate the scales or use the same models across the country. Accordingly, both BMI measures and BMI-based risk scores for some individuals may have been under- or overestimated. This, in turn, may have increased or decreased the observed differences between their BMI-based and cholesterol-based risk scores. However, BMI measurement errors were likely randomly distributed across the country and were not likely related to the measurement of any other variables used in the risk score calculations. Therefore, while the specific impact of BMI measurement error on the agreement between the two scores is unknown, it is unlikely that it contributed to any systematic differences between the BMI-based and the cholesterol-based CVD risk scores.

Practical considerations also led us to measure BP only once among participants for whom the first reading was considered normal, whereas we took the average of the final five BPTru readings for all others. While we may have overestimated BP among participants with a single reading (since BP readings tend to decrease with serial BPTru measures [23]), this overestimation would have had a similar impact on both CVD risk scores (i.e., the same BP was used in the calculation of an individual’s BMI-based and cholesterol-based risk). So, while both risk scores may have been overestimated for these individuals, any systematic impact on the agreement between the two scores was unlikely.

Few publications have compared laboratory and non-laboratory based CVD risk scores, and to the best of our knowledge, none report on agreement among South Asians. D’Agostino et al [11] did not publish the agreement between the two scores that we used in our screening clinics. Using data from the First National Health and Nutrition Examination Survey (NHANES I) and the NHANES I Epidemiologic Follow-up Study (NHEFS) cohort in the United States, Gaziano et al [24] developed a laboratory-based model incorporating age, systolic BP, smoking status, TC, reported diabetes status, and current treatment for hypertension to predict first fatal and non-fatal cardiovascular events. They also developed a non-laboratory-based model, substituting BMI for TC. Both models demonstrated good predictive discrimination, but within-person agreement by risk category was not reported. A subsequent analysis [25] conducted in the NHANES III population compared the non-laboratory-based model developed in the NHANES I population with four laboratory-based risk scores [11,26,27]. With risk categories defined as low risk (< = 10%) versus high risk (>10%), agreement between the non-laboratory-based model and the Framingham score that we also used [11] was 93% for women and 92% for men, somewhat higher than the agreement we observed. This could in part be explained by the different risk categorizations used. Green et al [28] calculated the same two risk CVD risk prediction scores that we did using data from the electronic health records of a group health cooperative in the United States and found that risk categories (low, moderate and high) were concordant for 78% of patients, a finding comparable to ours. Similarly, BMI-based risk classifications also tended to be higher than cholesterol-based risk classifications. This was more pronounced than it was among our participants, with 20% and 1% of their patients having higher and lower BMI-based classifications, respectively, compared with cholesterol-based classifications.

We selected the Framingham 10-year general CVD risk score [11] because it offered information on the global risk of developing any major atherosclerotic CVD event, included a number of risk factors that we were able to collect information on, and offered simple, paper-based calculation tables for both cholesterol-based and BMI-based risk assessment. We recognize that risk tools developed and validated in one population may not accurately predict CVD risk in another population due to secular, geographical and ethnic differences. While Aarabi et al [29] developed a modified Framingham-based coronary heart disease (CHD) risk score specifically for use among SAs, the adjusted calculations were deemed too complicated for volunteers in the community-based screening setting. Further, the tool is limited to the prediction of CHD. Other risk calculators that include ethnicity (such as QRISK2 [30]), adjust for ethnicity post-hoc (such as JBS2 [31]), or have been calibrated for use in SA populations (such as ETHRISK [32]) either include variables that we were unable to assess or were again deemed difficult for our volunteers to use.

Perhaps most importantly, no published CVD risk prediction tool has been developed, validated or calibrated for any Canadian SA population. To do so would require population-based, longitudinal data on both risk factors and CVD events specific to SAs, data that are currently unavailable in Canada. We selected the two Framingham-based scores recognizing that they would provide general estimates of CVD risk, and we were careful to explain to participants that the extent to which the scores over- or underestimate risk for SAs in Canada is not known.

An important component of our screening program will be the evaluation of change in CVD risk scores from baseline to follow-up. Accordingly, we needed to ascertain whether or not the two scoring tools we used similarly classified participants. While differences between scores were acceptable at the aggregate level, our Bland-Altman plots and comparison of categorical agreement show that the two risk scores cannot be used interchangeably in our study population.

Further, in this analysis, we computed the two Framingham risk scores using the formulae as published [11]. In practice at our screening clinics, however, volunteers calculated less exact scores using the paper-based risk charts included in the same paper. We also employed simple recommendations in an effort to adjust risk scores for SA ethnicity by adding 10 years to each participant’s age [29] and doubling the final risk score for those with a family history of premature CVD in a first degree relative before the age of 60 [33]. Adding 10 years to age would have a variable impact on the differences between scores, depending upon the participant’s age. We noted that the average difference between the two scores was higher for older participants, so the calculated difference between scores for a given participant would depend upon her/his adjusted-age categorization in the paper-based risk calculation sheets. Doubling the final risk scores based on family history would double the observed difference between the two scores. For example, for a participant with a BMI-based risk score of 8% and a cholesterol-based risk score of 14%, the difference between the risk estimates is 6%. If both risks were doubled based on family history, the difference between the estimates would increase to 12%. This would be of little practical significance when original differences between the scores are small (i.e., plus/minus 1 or 2 percent), but of substantial importance for participants with relatively large differences between BMI-based and cholesterol-based risks.

In light of the observed differences between the two risk scores as calculated, combined with the implications of adapting our scores, evaluation of a participant’s change in CVD risk over time will necessitate use of the same risk score (i.e., either BMI-based or cholesterol-based) at all screening sessions. It will be important for both participants and volunteer counselors to understand that the CVD risk score is an estimate, and only and estimate, useful as part of a comprehensive risk factor assessment and risk reduction plan. As our SA community-based research partners consider the continuance of a simple and sustainable program, they will need to adopt a protocol that ensures consistent risk appraisal within individuals.

## Conclusion

Despite acceptable average differences in BMI-based vs. cholesterol-based Framingham ten-year general CVD risk scores in our SA convenience sample, only moderate agreement between the scores was found when risk was categorized as low, moderate or high. While disagreements occurred in both directions, BMI-based risk was assessed as higher than cholesterol-based risk more often than it was found to be lower. Accordingly, the two risk scores cannot be used interchangeably in our national, community-based screening program. Valid assessment of change in participants’ CVD risk over time will require that one or the other be used consistently within participants who attend repeated screening sessions. Evaluation of the accuracy of either risk score in our population will require population-based longitudinal cohort studies among SAs in Canada.

## References

[1] Canada Statistics. Leading causes of death in Canada, 2009 Ottawa: Health Statistics Division.

[2] Sheth T, Nair C, Nargundkar M, Anand S, Yusuf S. Cardiovascular and cancer mortality among Canadians of European, south Asian and Chinese origin from 1979 to 1993: an analysis of 1.2 million deaths. CMAJ. 1999;161:132–138. 10439820 PMC1230461

[3] Nijjar AP, Wang H, Quan H, Khan NA. Ethnic and sex differences in the incidence of hospitalized acute myocardial infarction: British Columbia, Canada 1995–2002. BMC Cardiovasc Disord. 2010;10:38 10.1186/1471-2261-10-38 20723259 PMC2933615

[4] Ramaraj R, Chellappa P. Cardiovascular risk in South Asians. Postgrad Med J. 2008;84:518–523. 10.1136/pgmj.2007.066381 19017836

[5] Jones CA, Mawani S, King KM, Allu SO, Smith M, Mohan S, et al Tackling health literacy: adaptation of public hypertension educational materials for an Indo-Asian population in Canada. BMC Public Health. 2011;11:24 10.1186/1471-2458-11-24 21223580 PMC3030537

[6] Patel M, Phillips-Caesar E, Boutin-Foster C. Barriers to lifestyle behavioral change in migrant South Asian populations. J Immigr Minor Health. 2012;14:774–785. 10.1007/s10903-011-9550-x 22180198 PMC4666510

[7] Lai DW, Surood S. Types and factor structure of barriers to utilization of health services among aging South Asians in Calgary, Canada. Can J Aging. 2010;29:249–258. 10.1017/S0714980810000188 20416125

[8] Jones CA, Nanji A, Mawani S, Davachi S, Ross L, Vollman A, et al Feasibility of community-based screening for cardiovascular disease risk in an ethnic community: the South Asian Cardiovascular Health Assessment and Management Program (SA-CHAMP). BMC Public Health. 2013;13:160 10.1186/1471-2458-13-160 23432996 PMC3614427

[9] Patel JV, Gunarathne A, Lane D, Lim HS, Tracey I, Panja NC, et al Widening access to cardiovascular healthcare: community screening among ethnic minorities in inner-city Britain—the Healthy Hearts Project. BMC Health Serv Res. 2007;7:192 18036225 10.1186/1472-6963-7-192 PMC2222625

[10] Rao N, Eastwood SV, Jain A, Shah M, Leurent B, Harvey D, et al Cardiovascular risk assessment of South Asians in a religious setting: a feasibility study. Int J Clin Pract. 2012;66:262–269. 10.1111/j.1742-1241.2011.02773.x 22151579

[11] D’Agostino RB Sr, Vasan RS, Pencina MJ, Wolf PA, Cobain M, Massaro JM, et al General cardiovascular risk profile for use in primary care: the Framingham Heart Study. Circulation. 2008;117:743–753. 10.1161/CIRCULATIONAHA.107.699579 18212285

[12] Jones C, Simpson SH, Mitchell D, Haggarty S, Campbell N, Then K, et al Enhancing hypertension awareness and management in the elderly: lessons learned from the Airdrie Community Hypertension Awareness and Management Program (A-CHAMP). Can J Cardiol. 2008;24:561–567. 18612498 10.1016/s0828-282x(08)70634-2 PMC2640333

[13] Davachi S, Flynn M, Edwards A. A health region/community partnership for type 2 diabetes risk factor screening in Indo-Asian communities. Can J Diabetes. 2005;13:87–94.

[14] Robinson Vollman A, Anderson ET, McFarlane J, editors. Canadian community as partner: theory & multidisciplinary practice. 2nd ed. Philadelphia: Lippincott Williams & Wilkins; 2008 10.3109/01612840.2012.714054

[15] Allison C. BpTRU(tm) blood pressure monitor for use in a physician’s office. Issues Emerg Health Technol. 2006:1–4. 16958188

[16] Myers MG, Valdivieso M, Kiss A. Consistent relationship between automated office blood pressure recorded in different settings. Blood Press Monit. 2009;14:108–111. 10.1097/MBP.0b013e32832c5167 19417634

[17] Quinn RR, Hemmelgarn BR, Padwal RS, Myers MG, Cloutier L, Bolli P, et al The 2010 Canadian Hypertension Education Program recommendations for the management of hypertension: part I—blood pressure measurement, diagnosis and assessment of risk. Can J Cardiol. 2010;26:241–248. 20485688 10.1016/s0828-282x(10)70378-0 PMC2886554

[18] Jain A, Persaud JW, Rao N, Harvey D, Robertson L, Nirmal L, et al Point of care testing is appropriate for National Health Service health check. Ann Clin Biochem. 2011;48:159–165. 10.1258/acb.2010.010195 21355015

[19] Parikh P, Mochari H, Mosca L. Clinical utility of a fingerstick technology to identify individuals with abnormal blood lipids and high-sensitivity C-reactive protein levels. Am J Health Promot. 2009;23:279–282. 10.4278/ajhp.071221140 19288850 PMC2750040

[20] Bland JM, Altman DG. Statistical methods for assessing agreement between two methods of clinical measurement. Lancet. 1986;1:307–310. 2868172

[21] Cyr L, Francis K. Measures of clinical agreement for nominal and categorical data: the kappa coefficient. Comput Biol Med. 1992;22:239–246. 1643847 10.1016/0010-4825(92)90063-s

[22] Altman DG. Practical statistics for medical research. London: Chapman and Hall; 1991.

[23] Bryan S, Saint-Pierre Larose M, Campbell N, Clarke J, Tremblay MS. Resting blood pressure and heart rate measurement in the Canadian Health Measures Survey, cycle 1. Health Rep. 2010;21:71–78. 20426229

[24] Gaziano TA, Young CR, Fitzmaurice G, Atwood S, Gaziano JM. Laboratory-based versus non-laboratory-based method for assessment of cardiovascular disease risk: the NHANES I Follow-up Study cohort. Lancet. 2008;371:923–931. 10.1016/S0140-6736(08)60418-3 18342687 PMC2864150

[25] Pandya A, Weinstein MC, Gaziano TA. A comparative assessment of non-laboratory-based versus commonly used laboratory-based cardiovascular disease risk scores in the NHANES III population. PLoS One. 2011;6:e20416 10.1371/journal.pone.0020416 21655241 PMC3105026

[26] Anderson KM, Odell PM, Wilson PW, Kannel WB. Cardiovascular disease risk profiles. Am Heart J. 1991;121:293–298. 1985385 10.1016/0002-8703(91)90861-b

[27] Conroy RM, Pyorala K, Fitzgerald AP, Sans S, Menotti A, De Backer G, et al Estimation of ten-year risk of fatal cardiovascular disease in Europe: the SCORE project. Eur Heart J. 2003;24:987–1003. 12788299 10.1016/s0195-668x(03)00114-3

[28] Green BB, Anderson ML, Cook AJ, Catz S, Fishman PA, McClure JB, et al Using body mass index data in the electronic health record to calculate cardiovascular risk. Am J Prev Med. 2012;42:342–347. 10.1016/j.amepre.2011.12.009 22424246 PMC3308122

[29] Aarabi M, Jackson PR. Predicting coronary risk in UK South Asians: an adjustment method for Framingham-based tools. Eur J Cardiovasc Prev Rehabil. 2005;12:46–51. 15703505

[30] Hippisley-Cox J, Coupland C, Vinogradova Y, Robson J, Minhas R, Sheikh A, et al Predicting cardiovascular risk in England and Wales: prospective derivation and validation of QRISK2. BMJ. 2008;336:1475–1482. 10.1136/bmj.39609.449676.25 18573856 PMC2440904

[31] JBS 2:. Joint British Societies’ guidelines on prevention of cardiovascular disease in clinical practice. Heart. 2005;91 Suppl 5:v1–52. 16365341 10.1136/hrt.2005.079988 PMC1876394

[32] Brindle P, May M, Gill P, Cappuccio F, D’Agostino R Sr, Fischbacher C, et al Primary prevention of cardiovascular disease: a web-based risk score for seven British black and minority ethnic groups. Heart. 2006;92:1595–1602. 16762981 10.1136/hrt.2006.092346 PMC1861244

[33] Genest J, McPherson R, Frohlich J, Anderson T, Campbell N, Carpentier A, et al 2009 Canadian Cardiovascular Society/Canadian guidelines for the diagnosis and treatment of dyslipidemia and prevention of cardiovascular disease in the adult-2009 recommendations. Can J Cardiol. 2009;25:567–579. 19812802 10.1016/s0828-282x(09)70715-9 PMC2782500

